# An assessment of temporal, spatial and taxonomic trends in harmful algal toxin exposure in stranded marine mammals from the U.S. New England coast

**DOI:** 10.1371/journal.pone.0243570

**Published:** 2021-01-06

**Authors:** Spencer E. Fire, Andrea Bogomolni, Robert A. DiGiovanni, Greg Early, Tod A. Leighfield, Keith Matassa, Glenn A. Miller, Kathleen M. T. Moore, Michael Moore, Misty Niemeyer, Katie Pugliares, Zhihong Wang, Frederick W. Wenzel

**Affiliations:** 1 Florida Institute of Technology, Melbourne, FL, United States of America; 2 Massachusetts Maritime Academy, Buzzards Bay, Massachusetts, United States of America; 3 Atlantic Marine Conservation Society, Hampton Bays, New York, United States of America; 4 Integrated Statistics, Woods Hole, Massachusetts, United States of America; 5 National Oceanic and Atmospheric Administration, National Ocean Service, Charleston, South Carolina, United States of America; 6 Ocean Animal Response and Research Alliance, Laguna Niguel, California, United States of America; 7 International Fund for Animal Welfare, Yarmouth Port, Massachusetts, United States of America; 8 Biology Department, Woods Hole Oceanographic Institution, Woods Hole, Massachusetts, United States of America; 9 New England Aquarium, Boston, Massachusetts, United States of America; 10 CSS Corporation, Fairfax, VA, United States of America; 11 Under Contract to National Ocean Service, Charleston, South Carolina, United States of America; 12 National Oceanic and Atmospheric Administration, National Marine Fisheries Service, Woods Hole, Massachusetts, United States of America; University of Connecticut, UNITED STATES

## Abstract

Despite a long-documented history of severe harmful algal blooms (HABs) in New England coastal waters, corresponding HAB-associated marine mammal mortality events in this region are far less frequent or severe relative to other regions where HABs are common. This long-term survey of the HAB toxins saxitoxin (STX) and domoic acid (DA) demonstrates significant and widespread exposure of these toxins in New England marine mammals, across multiple geographic, temporal and taxonomic groups. Overall, 19% of the 458 animals tested positive for one or more toxins, with 15% and 7% testing positive for STX and DA, respectively. 74% of the 23 different species analyzed demonstrated evidence of toxin exposure. STX was most prevalent in Maine coastal waters, most frequently detected in common dolphins (*Delphinus delphis*), and most often detected during July and October. DA was most prevalent in animals sampled in offshore locations and in bycaught animals, and most frequently detected in mysticetes, with humpback whales (*Megaptera novaeangliae*) testing positive at the highest rates. Feces and urine appeared to be the sample matrices most useful for determining the presence of toxins in an exposed animal, with feces samples having the highest concentrations of STX or DA. No relationship was found between the bloom season of toxin-producing phytoplankton and toxin detection rates, however STX was more likely to be present in July and October. No relationship between marine mammal dietary preference and frequency of toxin detection was observed. These findings are an important part of a framework for assessing future marine mammal morbidity and mortality events, as well as monitoring ecosystem health using marine mammals as sentinel organisms for predicting coastal ocean changes.

## Introduction

Harmful algal blooms (HABs) are a major health threat to marine wildlife, especially marine mammals that inhabit waters where such blooms are common. The primary risk associated with HAB exposure in marine mammals is the production of natural toxins by various species of phytoplankton, and the subsequent concentration and accumulation of these toxins in marine mammal food webs [1, 2]. Certain HAB species produce potent neurotoxins that cause neurological symptoms which can lead to acute illness and/or death in mammals, such as decreased sensory function, seizures, ataxia, muscle incoordination, and respiratory paralysis [3–6]. During severe HAB events, the large geographic distribution of toxic phytoplankton and resultant widespread prevalence of these toxins throughout marine mammal food webs frequently results in mass mortality events (MMEs), sometimes involving multiple species of marine mammals.

In the coastal waters of the U.S., there are three groups of HAB toxins of primary concern due to their long history of negative impacts on coastal ecosystems and human health, namely brevetoxins, domoic acid (DA) and saxitoxins (STX). Each of these toxins has been associated with multiple human seafood poisoning events and frequent contamination of shellfish resources in the regions where their corresponding phytoplankton species are abundant. For example, brevetoxins comprise a suite of neurotoxins that causes the human illness known as neurotoxic shellfish poisoning (NSP), and is frequently associated with fish kills, human respiratory irritation and negative wildlife impacts from Texas to Florida [7–9]. Widespread brevetoxin accumulation in marine mammals occurs throughout this region [10–12] and has been linked to several large-scale MMEs dating back to the 1940s, with the number of marine mammal deaths associated with these events estimated at over 2200 animals [1, 13]. DA is a neurotoxin that is responsible for the human illness known as amnesic shellfish poisoning (ASP) and is responsible for frequent closures of shellfish beds and other commercial seafood resources [14, 15]. Although DA was first described as a clinical entity on the Atlantic coast of Canada and is present in nearly all U.S. coastal waters, its major impacts in North America are primarily observed from southern California to Washington, where high DA concentrations are frequently detected in marine mammals associated with seasonal HAB production [15–17]. DA exposure has similarly resulted in severe marine mammal MMEs along the Pacific U.S. coast, with estimated DA-related deaths possibly exceeding 10,000 animals over the last two decades [1, 4, 13, 18, 19; T. Norris pers. comm.].

STX is also a group of neurotoxins produced by HABs and causes the human illness known as paralytic shellfish poisoning (PSP), with symptoms that include loss of sensory input, ataxia, death by respiratory paralysis [20–23]. STX has been reported in varying degrees in several U.S. states, and its major impacts occur in New England coastal waters, where it has had devastating impacts on commercial fisheries and is a significant human health risk [24, 25]. STX accumulates in organisms from multiple trophic levels of the food web in this region, and has been associated with mortalities of various marine taxa, including finfish and seabirds [26–28]. However, unlike brevetoxins in Florida and DA in California, marine mammal MMEs caused by STX exposure in New England waters are rare, with the only documented case being a humpback whale die-off (n = 14 animals) in Cape Cod Bay, Massachusetts in 1987, and one unconfirmed event involving baleen whale mortalities (n = 21 animals) in the Gulf of Maine in 2003 [13, 29]. During 2005–2006, widespread closures of STX-contaminated shellfish beds throughout the Gulf of Maine co-occurred with 3 MMEs involving large whales and/or pinnipeds, but the causes of the MMEs were listed by resource managers as “Undetermined” or were attributed to infectious disease [13, 25]. This apparent lack of observed STX impacts on marine mammals is somewhat paradoxical, given 1) the extreme toxicity of STX relative to brevetoxins and DA (mouse i.p. LD_50_ 0.008 vs. 0.2 and 4.0 mg/kg b.w., respectively), 2) its widespread prevalence throughout the food web, and 3) the frequent, nearly annual STX-producing blooms occurring in the region [30–34].

The present study documents the degree of STX accumulation in a wide variety of marine mammal species recovered from New England coastal waters. The prevalence of DA exposure to the same marine mammal populations as a toxin of emerging concern in this region is also documented. Since HAB toxin-producing blooms are variable in time and space, a number of seasonal, temporal or geographic trends were assessed with regards to toxin exposure. In addition, this study investigated the possibility of differential toxin exposure based on the taxonomic groups present in our sample set. Further, since prey ingestion is the only biologically relevant route of DA or STX exposure for marine mammals, variability in toxin exposure as a factor of dietary habits was investigated. The findings from this investigation will help to resolve a critical need in seeking to establish causes for marine mammal MMEs in the future, since the source of many large-scale die-offs remains inconclusive and understanding of the role of HAB toxins in marine mammal deaths in this region is lacking. In addition, knowledge on the frequency of marine mammal exposure to either of these potent neurotoxins (DA and/or STX) will inform future studies aimed at assessing potential health risks to living marine mammal populations and their ability to thrive in a habitat where HABs are common.

## Methods

### Sample collection

**Fig 1:**
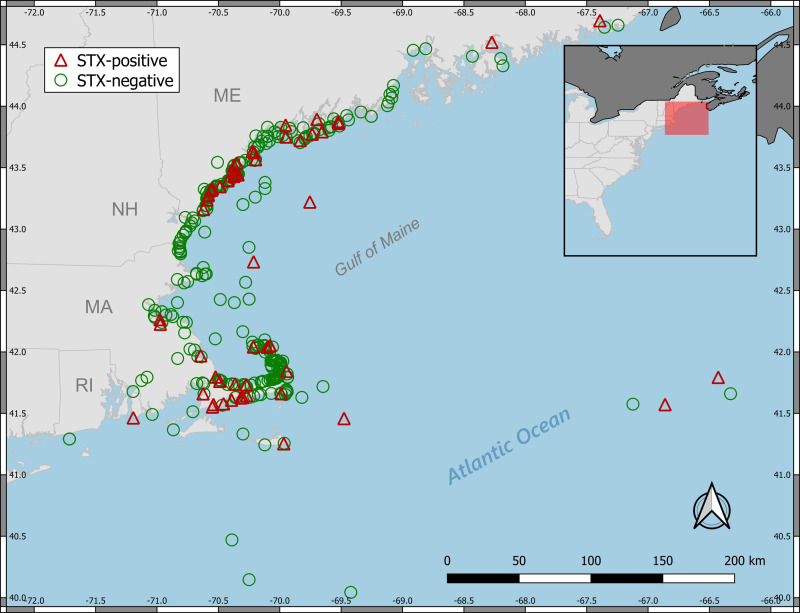
Animal collection/sampling locations and detections of saxitoxin (presence/absence in at least one sample).

**Fig 2:**
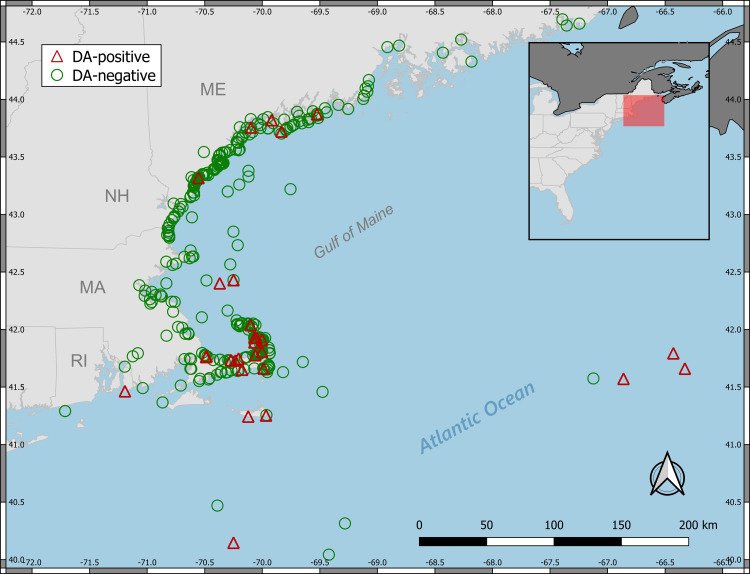
Animal collection/sampling locations and detections of domoic acid (presence/absence in at least one sample).

All work was performed under National Marine Fisheries Service Marine Mammal Health and Stranding Response Program Permit No. 082589. The study area comprised the coastal waters of the northeast Atlantic region of the United States (“New England” hereafter). The study area consisted of approximately 5500 linear km of coastline, with animals recovered mainly from beaches and nearshore waters (<1 km from shore). The majority of animals included in this study were either carcasses stranding perimortem or live animals euthanized shortly after response by trained NOAA marine mammal stranding network personnel. A small proportion (3.5%) of individuals analyzed were dead, fishery-bycaught animals opportunistically retained by the NOAA Northeast Fisheries Observer Program (NEFOP) at various offshore locations outside of state waters and subsequently delivered to the laboratory for necropsy and tissue sampling. All recovered carcasses were processed by trained and authorized stranding response staff following established federal protocols for cetacean necropsy and toxicological sampling [35]. Standardized data collection for each individual included stranding date, stranding location, state of decomposition, morphometrics, sex, age class, gross pathology, and evidence of human interaction. A total of 458 individual marine mammals were represented in the dataset across 4 U.S. states, including Massachusetts (n = 228), Maine (n = 199), New Hampshire (n = 10), and Rhode Island (n = 1). Bycaught animals and those collected well away from the coastline (e.g. Georges Bank) were assigned to a fifth region designated herein as “offshore/bycatch” (n = 20). The location of each animal’s stranding and/or sampling site are shown in Figs 1 and 2.

Of the 458 individual animals sampled, 271 (59%) were phocids, 144 (31%) were odontocetes, and 43 (9%) were mysticetes (a full dataset is included in S1 Table). In order to maximize the number of analyses available for the dataset, all existing relevant samples were analyzed, and therefore some species are underrepresented relative to those more commonly found stranded within the study area. In total, 23 marine mammal species were included in this study (plus one unidentified balaenopterid), with the number of individuals from each species listed in Table 1. Marine mammals collected and sampled between 1976 and 2016 were used as the source material for toxin analyses in the present study. Although the complete date range of sampled animals occurs over a 30-year time frame, nearly all (>98%) individuals were recovered after 2002.

**Table 1:** Number of sampled animals by common name and species.

Common name	Species name	n
Atlantic white-sided dolphin	*Lagenorhynchus acutus*	21
Bottlenose dolphin	*Tursiops truncatus*	6
Dwarf sperm whale	*Kogia sima*	1
Fin whale	*Balaenoptera physalus*	4
Gray seal	*Halichoerus grypus*	14
Harbor porpoise	*Phocoena phocoena*	17
Harbor seal	*Phoca vitulina*	210
Harp seal	*Pagophilus groenlandicus*	42
Hooded seal	*Cystophora cristata*	4
Humpback whale	*Megaptera novaeangliae*	12
Long-finned pilot whale	*Globicephala melas*	9
North Atlantic right whale	*Eubalaena glacialis*	2
Northern minke whale	*Balaenoptera acutorostrata*	23
Pygmy sperm whale	*Kogia breviceps*	4
Ringed seal	*Pusa hispida*	1
Risso's dolphin	*Grampus griseus*	8
Sei whale	*Balaenoptera borealis*	1
Short-beaked common dolphin	*Delphinus delphis*	70
Short-finned pilot whale	*Globicephala macrorhynchus*	2
Sowerby's beaked whale	*Mesoplodon bidens*	2
Sperm whale	*Physeter macrocephalus*	1
Striped dolphin	*Stenella coeruleoalba*	1
Whale, Unidentified Baleen	-	1
White-beaked dolphin	*Lagenorhynchus albirostris*	2

The biological samples (n = 916) collected from the various marine mammals described above were used as the source material for all toxin analyses in the present study. Samples collected for chemical analysis targeted organs or compartments relevant to dietary exposure, accumulation, and/or detoxification of HAB toxins, and were primarily composed of feces, gastric contents, urine and liver. However, in an effort to increase sample size, several other opportunistically collected sample types were also analyzed. A total of 13 different sample matrices were included in the toxin analyses, and the numbers of samples from each sample type are listed in Table 2.

**Table 2:** Distribution of sample types collected and analyzed for toxins.

Sample type	n
Feces	343
Gastric	279
Urine	170
Liver	72
Kidney	24
Bile	14
Aqueous humor	9
Serum	2
Abdominal contents	1
Amniotic fluid	1
Cardiac fluid	1
Milk	1
Pancreatic fluid	1

### Toxin extraction and analysis

Samples used in STX analyses were extracted according to methods described in Fire *et al*. [36]. Briefly, fluid samples were thawed, centrifuged at 10,000 x *g*, and filtered (0.45 μm) prior to analysis. For solid gastrointestinal contents and tissues, samples were extracted by homogenization in 80% acetonitrile/water (4:1, v/v) with 0.1% formic acid, and probe sonicated for 2 minutes. Homogenates were centrifuged at 3400 x g, and the supernatants were filtered (0.45 μm) and stored at -20°C prior to analysis. Samples were analyzed using receptor binding assay (RBA) and/or enzyme-linked immunosorbent assay (ELISA) methods, following procedures outlined in Van Dolah *et al*. [37] and Fire *et al*. [10], respectively. The RBA measures competition between radiolabeled STX and STX in the sample or in standards (NIST RM 8642) for binding to the voltage-gated sodium channel, the pharmacological target of STX, to determine the total STX-like activity of the sample. The RBA detection limit was approximately 3.0 ng STX equivalents/g for solid samples and 1.0 ng STX equivalents/mL for fluid samples. ELISA methods used a commercially available, direct competitive STX kit (Biosense Laboratories, Bergen, Norway) following protocols outlined by the manufacturer. The assay measures STX in a sample extract via competition with a STX standard coated onto the microplate wells for anti-STX antibodies in solution. Extracts were typically diluted at 1:50 as part of the ELISA and RBA protocols. The limit of detection for the STX ELISA was approximately 1.0 ng STX/mL for fluid samples and 2.5 ng STX/mL for solid samples.

Samples used in DA analyses were extracted according to methods described in Fire *et al*. [38]. Briefly, fluid samples were thawed, centrifuged at 10,000 x *g* and filtered (0.45 μm) prior to analysis. For solid gastrointestinal contents and tissues, samples were extracted by homogenization in 50% aqueous methanol (1:4, w:v) and probe sonicated for 2 minutes. Homogenates were centrifuged at 3,400 x *g*, the supernatants were filtered (0.45 μm), purified using 200 mg C18 solid-phase extraction (SPE) columns, and were stored at –20°C prior to analysis. Extracts were analyzed for DA using ELISA and/or liquid chromatography tandem mass spectrometry (LC-MS/MS) methods, following procedures outlined in Maucher & Ramsdell [39] and Wang *et al*. [40], respectively. ELISA methods used a commercially available, direct competitive DA kit (Biosense Laboratories, Bergen, Norway) following protocols outlined by the manufacturer. This assay measures DA in a sample extract via competition with a DA standard coated onto the microplate wells for anti-DA antibodies in solution. Extracts were typically diluted at 1:100 as part of the ELISA protocol. The limit of detection of the DA ELISA was approximately 1.0 ng DA/mL for fluid samples and 2.5 ng DA/mL for solid samples. LC-MS/MS methods utilized reversed phase chromatography, using an Agilent Technologies 1100 HPLC (Agilent Technologies, USA) coupled to an AB SCIEX API-4000 (AB Sciex, USA) triple quadrupole mass spectrometer in ESI+ mode. Chromatographic separation was performed on a Phenomenex Luna C18(2), 5 μm, 150 mm x 2 mm column. The mobile phase consisted of water and acetonitrile in a binary system, with 0.1% formic acid as an additive. Retention time of DA in samples was determined based on the retention time observed with a certified DA reference standard (NRC, Halifax, Canada). The detection of DA by mass spectrometry was achieved by a Multiple Reaction Monitoring (MRM) method with Turboionspray interface. Four MRM transitions from protonated DA were monitored: m/z 312 → 266, m/z 312 → 248, m/z 312 → 193, and m/z 312 → 161. The limit of detection of the LC-MS/MS method was 2.5 ng DA/g sample, with a signal to noise ratio of 3 or higher for the MRM confirmation channel m/z 312 → 161. The limit of quantitation (LOQ) was 5 ng DA/g with a signal-to-noise ratio of 10 for the MRM quantitation channel m/z 312 → 266.

### Data analysis

Associated metadata for each animal was used to carry out several analyses to test for relationships between toxin exposure data and any of the following: sampling location, taxonomic group or species, sample type, dietary preference, and sampling month, year, or seasonality of co-occurring toxin-producing blooms. Tests comparing sampling location were performed after assigning each animal to one of five categories based on the sub-regions within our study area, corresponding to nearshore coastal waters of Maine (ME), Massachusetts (MA), New Hampshire (NH), and Rhode Island (RI), and offshore/bycatch (OB). Tests comparing taxonomic groups were based on animals being categorized as Mysticetes, Odontocetes, or Pinnipeds. Tests for individual species used only those that had at least one positive and one negative animal for the specified toxin, to reduce potential biases from the many under-represented species in the dataset. Tests comparing body compartments were based on the sample type as the sampling unit. Only sample type categories with both positive and negative results for each toxin were utilized (feces, 236 samples; gastric, 220 samples; liver, 49 samples; urine, 131 samples). Only animals from which more than one sample was taken were included. Differences in proportion of positive results was used as the test statistic for pairwise randomization comparisons of each combination of body compartments. As all included animals contributed samples to more than one sample type, randomization was not performed among animals. Instead randomization only occurred among samples taken from the same animal. For animals in which all samples were positive or negative, no randomization was possible and their results were added to the appropriate totals. Five thousand permutations were used to build the random distributions. Actual and randomized results were squared to remove negative values and make the comparisons two-tailed. P-values were the number of squared permuted differences greater than or equal to the actual squared differences divided by the number of permutations. The Bonferroni adjusted critical α for each comparison was 0.0083.

To test for associations between toxin exposure and dietary preference, we broadly categorized each species by its relative trophic level to the other species in the dataset for subsequent analyses. Classifications of major prey items followed descriptions of marine mammal diets and feeding habits as referenced in the literature [41–49], Wenzel et al., unpub.). The predominant prey organism(s) of each marine mammal species was considered, and each animal in the dataset was grouped into one of three broad categories: Group 1 = lower-trophic level (e.g. zooplankton), Group 2 = mid-trophic level (e.g. planktivorous/forage fishes), Group 3 = upper-trophic level (e.g. piscivorous fish/cephalopods) roughly corresponding to increasing trophic level of the corresponding prey in the food web (Table 5; [50, 51]).

Tests for temporal trends were based on the date each animal was first reported stranded, with the assumption that feeding events (and therefore dietary toxin exposure) would cease at the time of stranding. In addition, each animal was categorized as belonging to one of two sampling ‘seasons’, corresponding to the typical date ranges when HAB toxins are most likely present in the study area. The typical growing period for *Alexandrium fundyense* (the causative organism of STX-producing blooms in New England) is April through August, and is closely coupled to corresponding STX toxicity in the food web (e.g. shellfish) in the same region [52, 53]. Therefore, animals were assigned to the “during bloom season” (DBS) or “non-bloom season” (NBS) categories based on whether their reported stranding date was during April-August, or September-March, respectively, and comparative analysis of STX detection frequencies between these groups was performed. Insufficient data was available to determine seasonality of DA-producing blooms in the study area, thus similar comparisons of DA detection frequencies were not attempted.

Logistic regression was performed for tests comparing sampling locations, taxonomic groupings, species, diet, sampling month, sampling year, and bloom season. The response variable for all logistic regressions was the number of animals testing positive/negative for the stated toxin, either for saxitoxin (STX), domoic acid (DA), or for the presence of either toxin (DA and/or STX in the same animal). General comparisons for the ‘either toxin’ group were only performed if both the DA and STX tests were not statistically significant. For the categorical predictor variables of sampling location, taxonomic grouping, species, diet, sampling month, and bloom season, dummy variables corresponding to the levels of the stated variable were used as predictor variables. Sampling year was the only continuous predictor variable.

In addition to logistic regression, correspondence analysis (CA) was performed for each categorical predictor variable with more than two levels prior to the logistic regression analysis. CA examines the inertia (i.e. interdependence or lack of independence) between the levels of two variables expressed in a contingency table. The rows of the contingency table represent one variable and the columns represent the second variable. Each CA had four column variables: DA positive, DA negative, STX positive, and STX negative. The CA row variables were the levels of the indicated categorical predictor variable. The inertia is expressed in dimensions where the early dimensions explain more of the interdependent relationship between the variables than latter dimensions and the dimensions are uncorrelated with each other. High correlation between levels of the variables and a dimension indicate that the dimension explains much of the variation within those levels. High contribution of levels of the variables to a dimension indicates that the levels most represent the dimension. The CA ordination plots display the dimensions, as axes, and levels of one or both variables, as points. Levels located closer together have more in common than those farther apart. CA was not performed for predictor variables with only two levels as the results would be one-dimensional and not informative. The dummy variable in the logistic regression analyses was the level of the categorical predictor variable closest to the origin of the CA ordination. A dummy variable in logistic regression analysis is the level of the predictor variable represented by the intercept of the logistic regression equation. As the goal of the analyses with categorical predictor variables was not to generate a predictive model, the logistic regression equations are not reported but are instead used to generate the reported odds ratios. Significance levels for all logistic regression analyses were set at α = 0.05. CA does not include a significance test. Analyses were conducted in R 3.6.3 [54] and the package ca [55].

## Results

Overall, 85 (18.6%) of the 458 animals in our sample set tested positive for HAB toxins. Although a small number of samples had insufficient material to extract for both toxins, a total of 447 (97%) were tested for STX and 435 (95%) animals were tested for DA. Of the 1686 HAB toxin analyses performed from multiple sample matrices, 863 were for STX and 823 were for DA. We detected STX in 73 of 863 (8.5%) samples and DA in 33 of 823 (4.0%) samples. This corresponds to detection of STX in 65 of 447 (14.5%) animals and DA in 30 of 435 (6.9%) of animals sampled, with 10 (2.2%) individuals positive for both STX and DA.

### Geographic trends

The vast majority of animals tested were from MA and ME, and the proportion of HAB toxin-positive animals for these states were comparable, with 16.2% (40 of 199) and 20.1% (37 of 228) of individuals testing positive, respectively (Table 3). None of the 10 NH animals tested positive for either HAB toxin, and the single RI individual tested positive for both toxins. OB animals tested positive for the presence of either toxin in 35% (7 of 20) cases. In terms of STX exposure only, the proportion of STX-positive animals in MA and ME was 11.3% (24 of 218) and 18.6% (37 of 199), respectively. OB animals were STX-positive in 15.8% (3 of 19) of cases. For DA exposure, the proportion of DA-positive animals in MA and ME was 8.4% (18 of 214) and 2.6% (5 of 190), respectively). OB animals were DA-positive in 30% (6 of 20) of cases. More broadly, the spatial distribution of toxin-positive animals across the study area was somewhat heterogeneous, and appeared to be dependent on the toxin tested. STX-positive animals were widely distributed across the southern coast of Maine and along the eastern coastal regions of Massachusetts (Fig 1). In contrast, DA-positive animals were primarily concentrated on the Cape Cod peninsula of Massachusetts (Fig 2).

**Table 3:** Toxin detection frequencies by sub-region.

	Either toxin		STX		DA	
Region	n	%	n	%	n	%
Massachusetts (MA)	37/228	16.2%	24/218	11.0%	18/214	8.4%
Maine (ME)	40/199	20.1%	37/199	18.6%	5/190	2.6%
New Hampshire (NH)	0/10	0.0%	0/10	0.0%	0/10	0.0%
Rhode Island (RI)	1/1	100.0%	1/1	100.0%	1/1	100.0%
Offshore/bycatch (OB)	7/20	35.0%	3/19	15.8%	6/20	30.0%

RI was represented by a single animal and was omitted from statistical tests. NH was represented by animals testing negative only and was omitted from logistic regression but was included in the correspondence analysis. MA and ME were statistically different for frequencies of STX-positive animals: (p = 0.030), while OB was not statistically different from either MA or ME (p = 0.532). Animals in ME were 1.85 times more likely to test positive for STX compared to MA. For DA-positive animals, MA, ME, and OB all had statistically different frequencies of detection for DA (p < 0.02). OB animals were 15.86 times more likely to test DA-positive compared to ME, and 4.67 times more likely to test DA-positive compared to MA. Animals in the MA group were 3.40 times more likely to test DA-positive compared to ME.

**Fig 3:**
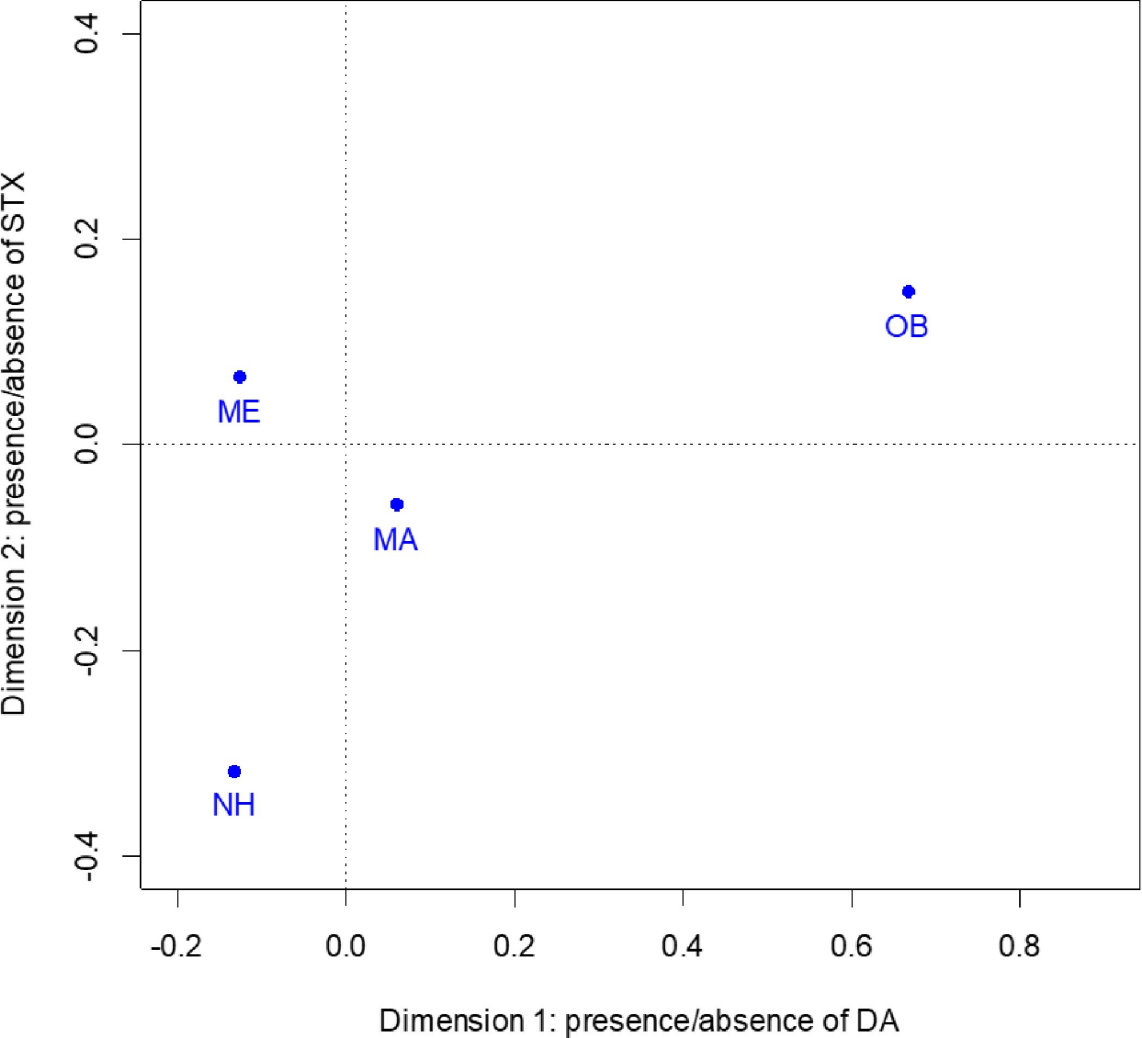
Correspondence analysis ordination for sub-region comparisons. Positive values for dimension 1 correlate with larger proportion of positive domoic acid samples and negative values correlate with larger proportion of negative domoic acid samples. Positive values for dimension 2 correlate with larger proportion of positive saxitoxin samples and negative values correlate with larger proportion of negative saxitoxin samples. MA = Massachusetts, ME = Maine, NH = New Hampshire, OB = offshore/bycatch.

CA dimension 1 explains 81.0% of the total inertia between toxins and location and correlates with DA positive/negative, 0.993 and 0.973 respectively. DA positive has the highest contribution, 922‰, to dimension 1. CA dimension 2 explains 19.0% of the total inertia between toxins and location and correlates with STX positive/negative, 0.858 and 0.972 respectively. STX positive has the highest contribution, 845‰, to dimension 2. MA, ME, and OB correlate with dimension 1, 0.521, 0.789, and 0.952 respectively, with OB having the highest contribution, 679‰. Only NH correlates with dimension 2, 0.846, and all locations show equivalent contributions. The CA ordination shows more separation among the locations in dimension 1 compared to dimension 2 (Fig 3).

### Taxonomic trends

Distribution of HAB toxin-positive animals by taxonomic group was as follows: phocids 18.5% (50 of 271), odontocetes 17.4% (25 of 144), and mysticetes 23.3% (10 of 43; Table 4). The highest frequency of detection among the more abundant species represented in the dataset (n ≥ 10 animals) was in humpback whales (33.3%), followed by gray seal (28.6%), northern minke whale (21.7%), and harp seal (21.4; Table 5). Distribution of STX-positive animals by taxonomic group was as follows: mysticetes 17.1% (7 of 41), phocids 16.4% (44 of 269), and odontocetes 10.2% (14 of 137). By species, the highest frequency of STX detection (n ≥ 10 animals) was in harp seal (19%), followed by northern minke whale (18%), humpback whale (18%), harbor seal (15.8%), gray seal (14.3%) and harbor porpoise (13.3%). Distribution of DA-positive animals by taxonomic group was as follows: mysticetes 19% (8 of 42), odontocetes 10.6% (14 of 132), and phocids 3.1% (8 of 261). By species, the highest frequency of DA detection (n ≥ 10 animals) was in humpback whales (33.3%), followed by northern minke whale (17%), gray seal (15.4%), and short-beaked common dolphin (12.7%).

**Table 4:** Toxin detection frequencies by taxonomic group.

	Either toxin		STX		DA	
Taxonomic Group	n	%	n	%	n	%
Phocids	50/272	18.4%	44/269	16.4%	8/261	3.1%
Odontocetes	25/145	17.2%	14/137	10.2%	14/132	10.6%
Mysticetes	10/43	23.3%	7/41	17.1%	8/42	19.0%

**Table 5:** Toxin detection frequencies by species.

			*Prey	Either toxin		STX		DA	
Species name	Common name	n	Group	pos./n	%	pos./n	%	pos./n	%
*Phoca vitulina*	Harbor seal	210	2	36/210	17.1%	33/209	15.8%	4/203	2%
*Pagophilus groenlandicus*	Harp seal	42	3	9/42	21.4%	8/42	19%	2/42	4.8%
*Halichoerus grypus*	Gray seal	14	2	4/14	28.6%	2/14	14.3%	2/13	15.4%
*Cystophora cristata*	Hooded seal	4	3	1/4	25%	1/3	33%	0/2	0%
*Pusa hispida*	Ringed seal	1	3	0/1	0%	0/1	0%	0/1	0%
*Delphinus delphis*	Short-beaked common dolphin	70	3	8/70	11%	1/68	2%	8/64	12.5%
*Lagenorhynchus acutus*	Atlantic white-sided dolphin	21	3	1/21	4.8%	1/20	5%	0/20	0%
*Phocoena phocoena*	Harbor porpoise	17	3	2/17	11.8%	2/15	13.3%	0/16	0%
*Globicephala melas*	Long-finned pilot whale	9	3	1/9	11%	1/9	11%	1/9	11%
*Grampus griseus*	Risso's dolphin	8	3	5/8	63%	5/8	63%	0/8	0%
*Tursiops truncatus*	Bottlenose dolphin	6	3	1/6	16.7%	1/5	20%	0/5	0%
*Kogia breviceps*	Pygmy sperm whale	4	3	4/4	100%	0/4	0%	4/4	100%
*Mesoplodon bidens*	Sowerby's beaked whale	2	2	0/2	0%	0/2	0%	0/1	0%
*Lagenorhynchus albirostris*	White-beaked dolphin	2	3	0/2	0%	0/2	0%	0/1	0%
*Globicephala macrorhynchus*	Short-finned pilot whale	2	3	1/2	50%	1/2	50%	0/2	0%
*Kogia sima*	Dwarf sperm whale	1	3	1/1	100%	1/1	100%	1/1	100%
*Physeter macrocephalus*	Sperm whale	1	3	0/1	0%	0/1	0%	0/1	0%
*Stenella coeruleoalba*	Striped dolphin	1	3	1/1	100%	1/1	100%	0/1	0%
*Balaenoptera acutorostrata*	Northern minke whale	23	2	5/23	22%	4/22	18%	4/22	18.2%
*Megaptera novaeangliae*	Humpback whale	12	2	4/12	33.3%	2/11	18.2%	4/12	33.3%
*Balaenoptera physalus*	Fin whale	4	1	1/4	25%	1/4	25%	0/4	0%
*Eubalaena glacialis*	North Atlantic right whale	2	1	0/2	0%	0/2	0%	0/2	0%
*Balaenoptera borealis*	Sei whale	1	1	0/1	0%	0/1	0%	0/1	0%
*Unidentified Balaenopterid*	Whale, Unidentified Baleen	1	2	0/1	0%	0/1	0%	0/1	0%

*1 = lower-trophic level, 2 = mid-trophic level, 3 = upper-trophic level.

Logistic regression analysis comparing proportions of STX-positive animals by taxonomic group showed no significant differences for STX (p > 0.098). However, frequencies of DA-positive phocids, mysticetes and odontocetes were all statistically different (p < 0.04). Mysticetes were 7.44 times more likely to test positive for DA compared to phocids, and 1.98 times more likely to test positive compared to odontocetes. Odontocetes were 3.75 times more likely to test positive for DA relative to phocids.

**Fig 4:**
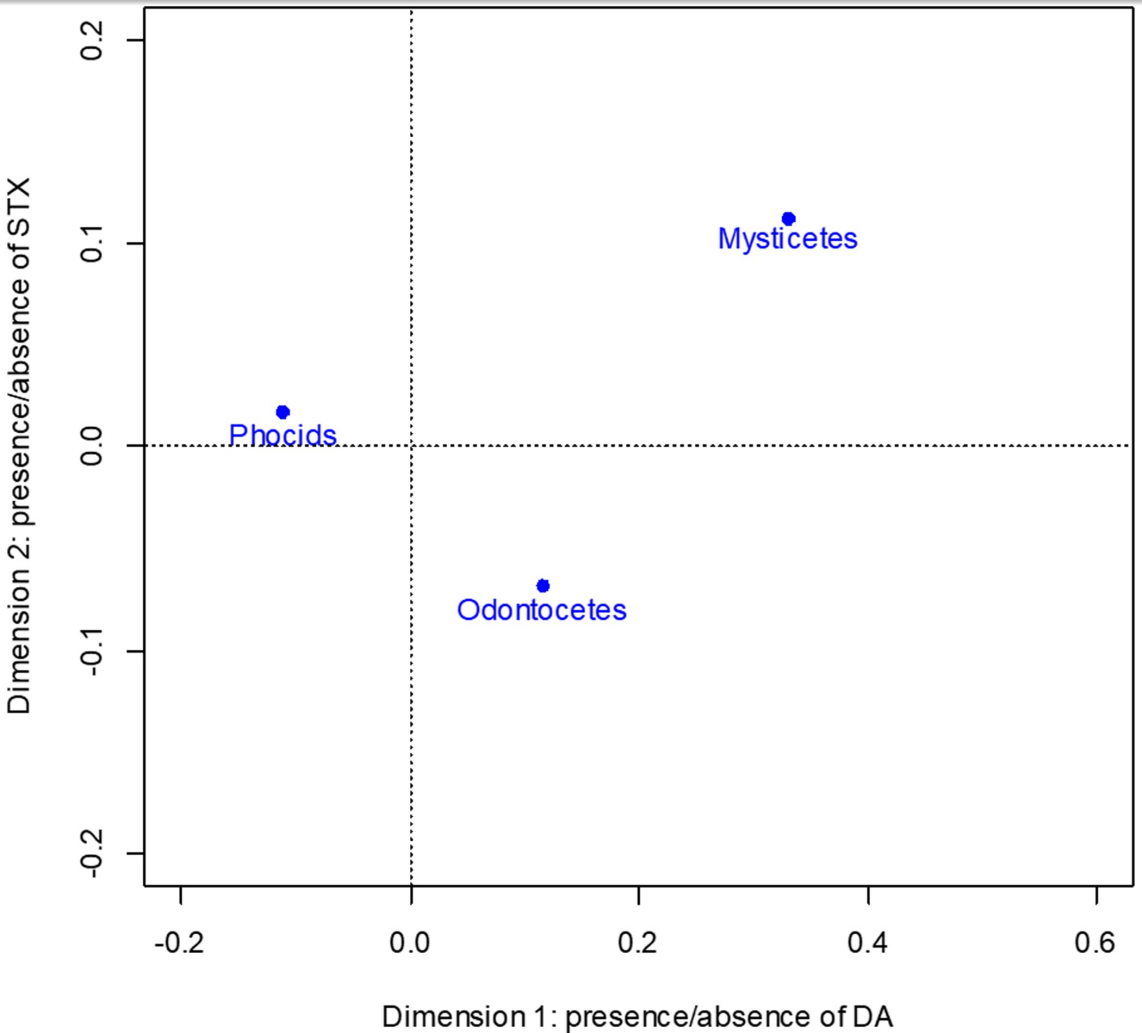
Correspondence analysis ordination for taxonomic group comparisons. Positive values for dimension 1 correlate with larger proportion of positive domoic acid samples and negative values correlate with larger proportion of negative domoic acidsamples. Positive values for dimension 2 correlate with larger proportion of positive saxitoxin samples and negative values correlate with larger proportion of negative saxitoxin samples.

CA dimension 1 explains 88.7% of the total inertia between toxins and taxonomic groups and correlates with DA positive/negative, 0.995, and 0.996 respectively. DA positive has the highest contribution, 910‰, to dimension 1. CA dimension 2 explains 11.3% of the total inertia between toxins and taxonomic group and correlates with STX positive/negative, 0.751 and 0.919 respectively. STX positive has the highest contribution, 752‰, to dimension 2. All taxonomic groups correlate with dimension 1, phocids 0.978, mysticetes 0.895, and odontocetes 0.746, and have equivalent contributions. None of the taxonomic groups correlate with dimension 2. The CA ordination shows more separation among the taxonomic groups in dimension 1 compared to dimension 2 (Fig 4).

Analysis comparing toxin-positive frequency by species included only those with ≥1 positive and ≥ 1 negative animal for each toxin, resulting in 7 species (*D*. *delphis*, *H*. *grypus*, *B*. *acutorostrata*, *M*. *novaeangliae*, *P*. *groenlandicus*, *P*. *vitulina*, *G*. *melas*) being included in the tests. The frequency of STX-positive *D*. *delphis* was significantly higher relative to *P*. *vitulina* (p = 0.014), *B*. *acutorostrata* (p = 0.019), *M*. *novaeangliae* (p = 0.035), and *P*. *groenlandicus* (p = 0.011), with *D*. *delphis* being 12.38, 14.67, 14.67 and 15.53 times more likely to test STX-positive, respectively. The frequency of DA-positive *P*. *vitulina* was significantly lower than *D*. *delphis* (p = 0.002), *H*. *grypus* (p = 0.017), *B*. *acutorostrata* (p = 0.001), and *M*. *novaeangliae* (p < 0.001), with *P*. *vitulina* being 7.24, 9.05, 11.06 and 24.88 times less likely to test DA-positive than the comparison species. The frequency of DA-positive *M*. *novaeangliae* was also significantly higher than *P*. *groenlandicus* (p = 0.015) and *P*. *vitulina* (p < 0.001), with *M*. *novaeangliae* 10.00 and 24.88 times more likely to test DA-positive, respectively.

**Fig 5:**
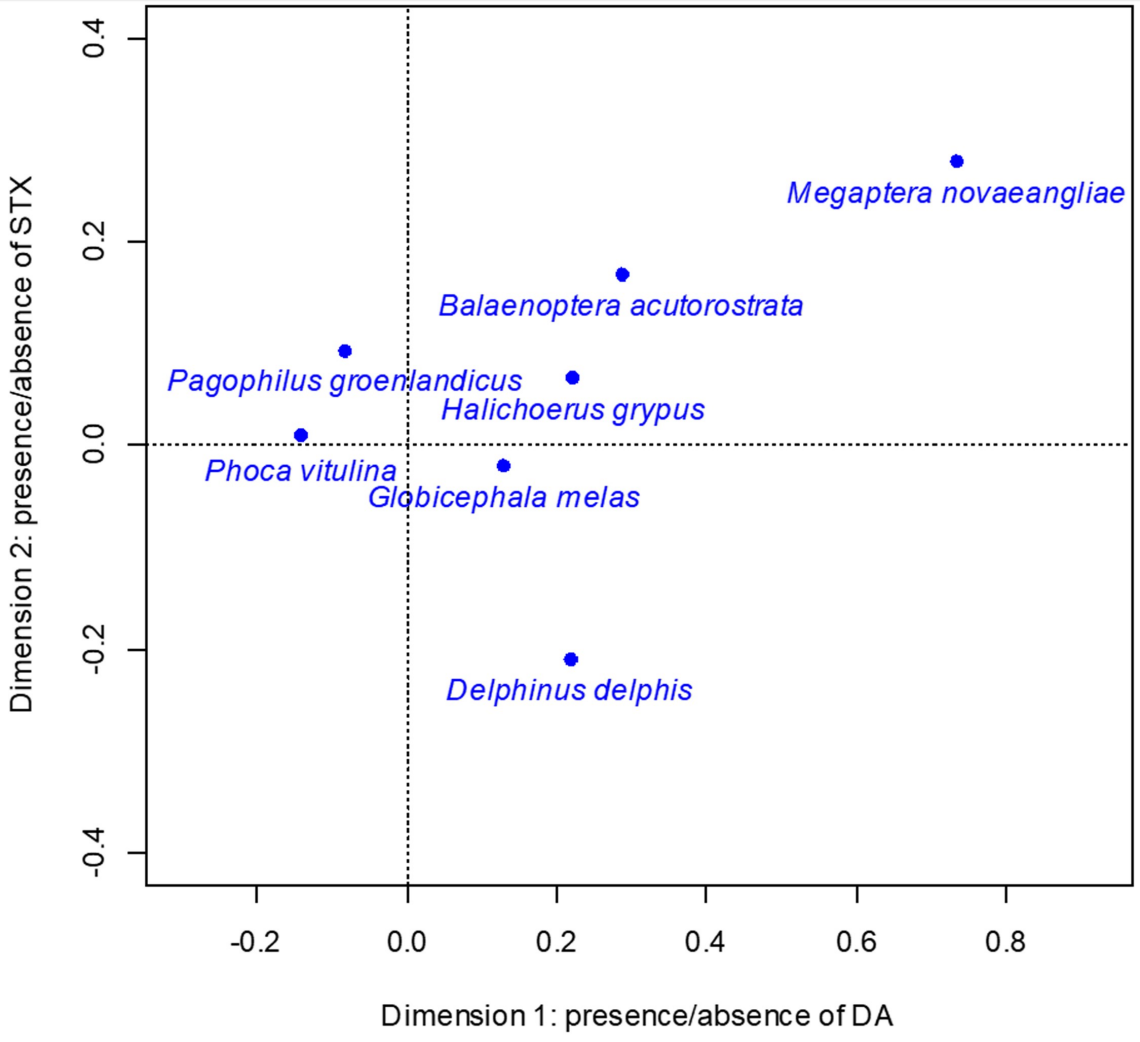
Correspondence analysis ordination for species comparisons. Positive values for dimension 1 correlate with larger proportion of positive domoic acid samples and negative values correlate with larger proportion of negative domoic acid samples. Positive values for dimension 2 correlated with larger proportion of positive saxitoxin samples and negative values correlate with larger proportion of negative saxitoxin samples.

CA dimension 1 explains 63.7% of the total inertia between toxins and species and correlates with DA positive/negative, 0.979 and 0.988 respectively. DA positive has the highest contribution, 704‰, to dimension 1. CA dimension 2 explains 35.7% of the total inertia between toxins and species and correlates with STX positive/negative, 0.807 and 0.884 respectively. STX positive has the highest contribution, 626‰, to dimension 2. Table 6 summaries the correlations and contributions of all species included in the CA. The CA ordination shows more separation among species in dimension 1 compared to dimension 2. The CA ordination also displays species with increasing proportional instances of DA positive from left to right and increasing proportional instances of STX positive from bottom to top (Fig 5).

**Table 6:** Species correlations and contributions to correspondence analysis dimensions 1 and 2.

	Dimension 1		Dimension 2	
	Correlation	Contribution (‰)	Correlation	Contribution (‰)
*Balaenoptera acutorostrata*	0.743	0.110	0.257	0.128
*Delphinus delphis*	0.515	0.187	0.485	0.592
*Globicephala melas*	0.967	0.009	0.022	0.001
*Halichoerus grypus*	0.896	0.040	0.083	0.012
*Megaptera novaeangliae*	0.872	0.378	0.127	0.186
*Pagophilus groenlandicus*	0.446	0.018	0.552	0.076
*Phoca vitulina*	0.995	0.257	0.005	0.004

### Distribution of toxin in body compartments

HAB toxins were most frequently detected in feces (15.5%) and urine samples (14.7%), followed by liver (9.7%) and gastric contents (2.5%; Table 7). Although sample numbers were limited, bile samples also tested positive (28.6%), but these were only positive for STX analyses. None of the other sample types collected tested positive for either STX or DA. In terms of concentration of toxin detected, the highest maximum STX concentrations by sample type were present in feces (max. 1790 ng/g), followed by bile (max. 1224 ng/g), liver (max. 810 ng/g), urine (max. 304 ng/mL) and gastric contents (max. 200 ng/g). Similarly, the highest DA concentrations were detected in feces samples (max. 394 ng/g), followed by gastric contents (max. 200 ng/g), urine (12 ng/mL), and liver (6 ng/g). Constrained randomization analyses found that for STX, frequency of detection in gastric samples was significantly lower relative to feces (p < 0.001) and urine samples (p = 0.007). Similarly, for DA, the frequency of detection in gastric samples was significantly lower relative to feces (p = 0.002) and urine samples (p = 0.008).

**Table 7:** Toxin detection frequencies and toxin concentration ranges, grouped by sample type and toxin.

	Either toxin		STX					DA				
Sample type	n	%	n	%	min	avg	max	n	%	min	avg	Max
Feces	53/343	15.5%	41/327	12.5%	2.3	262	1790	19/303	6.3%	2	51.8	394
Gastric	7/279	2.5%	5/268	1.9%	7	88.2	200	3/253	1.2%	trace	-	200
Urine	25/170	14.7%	17/148	11.5%	0.7	70	304	10/158	6.3%	0.2	1.7	12
Liver	7/72	9.7%	6/71	8.5%	6	301.6	810	1/64	1.6%	6	6	6
Kidney	0/24	0.0%	0/23	0%	-	-	-	0/21	0%	-	-	-
Bile	4/14	28.6%	4/14	28.6%	256	581	1224	0/14	0%	-	-	-
Aqueous humor	0/9	0%	0/5	0%	-	-	-	0/5	0%	-	-	-
Serum	0/2	0%	0/2	0%	-	-	-	0/1	0%	-	-	-
Abdominal contents	0/1	0%	0/1	0%	-	-	-	0/1	0%	-	-	-
Amniotic fluid	0/1	0%	0/1	0%	-	-	-	0/1	0%	-	-	-
Cardiac fluid	0/1	0%	0/1	0%	-	-	-	0/1	0%	-	-	-
Milk	0/1	0%	0/1	0%	-	-	-	0/1	0%	-	-	-
Pancreatic fluid	0/1	0%	0/1	0%	-	-	-	0/1	0%	-	-	-

‘min’, ‘avg’, and ‘max’ = minimum, mean, and maximum concentrations, respectively, reported in nanograms of toxin per gram of sample.

### Influence of dietary preference on HAB toxin exposure

Species assigned to Prey Groups 2 (mid-trophic level prey) or 3 (upper-trophic level prey) had similar frequencies of testing positive for either HAB toxin (18.7% and 18.5%, respectively), while 14.3% of those assigned to Prey Group 1 (lower-trophic level prey) tested positive for either toxin (Table 8). For STX only, species assigned to Group 2 had the highest probability of testing positive for STX (15.8%), followed by Group 1 with 14.3% and Group 3 with 12.6%. For DA, Group 3 had the highest probability of testing positive for DA (9.0%), followed by Group 2 with 5.6%. None of the Group 1 animals tested positive for DA. Logistic regression analysis of toxin detections by prey group showed no significant differences in frequency of detection between groups for STX (p > 0.900), DA (p = 0.163), or either toxin (p > 0.700).

**Table 8:** Frequency of toxin-positive animals by dietary preference.

	Either toxin			STX			DA		
Prey Group	# tested	# pos.	%	# tested	# pos.	%	# tested	# pos.	%
1—Lower-trophic level	7	1	14.3%	7	1	14.3%	7	0	0.0%
2—Mid-trophic level	262	49	18.7%	259	41	15.8%	252	14	5.6%
3—Upper-trophic level	189	35	18.5%	182	23	12.6%	178	16	9.0%

CA dimension 1 explains 95.5% of the total inertia between toxins and dietary preference and correlates with all toxin variables: DA positive 0.988, DA negative 0.967, STX positive 0.849, STX negative 0.920. DA positive has the highest contribution, 720‰, to dimension 1. All dietary preferences correlate with dimension 1, Group 1 (0.753), Group 2 (0.986), and Group 3 (0.997), and have equivalent contributions.

### Temporal trends of HAB toxin exposure

**Fig 6:**
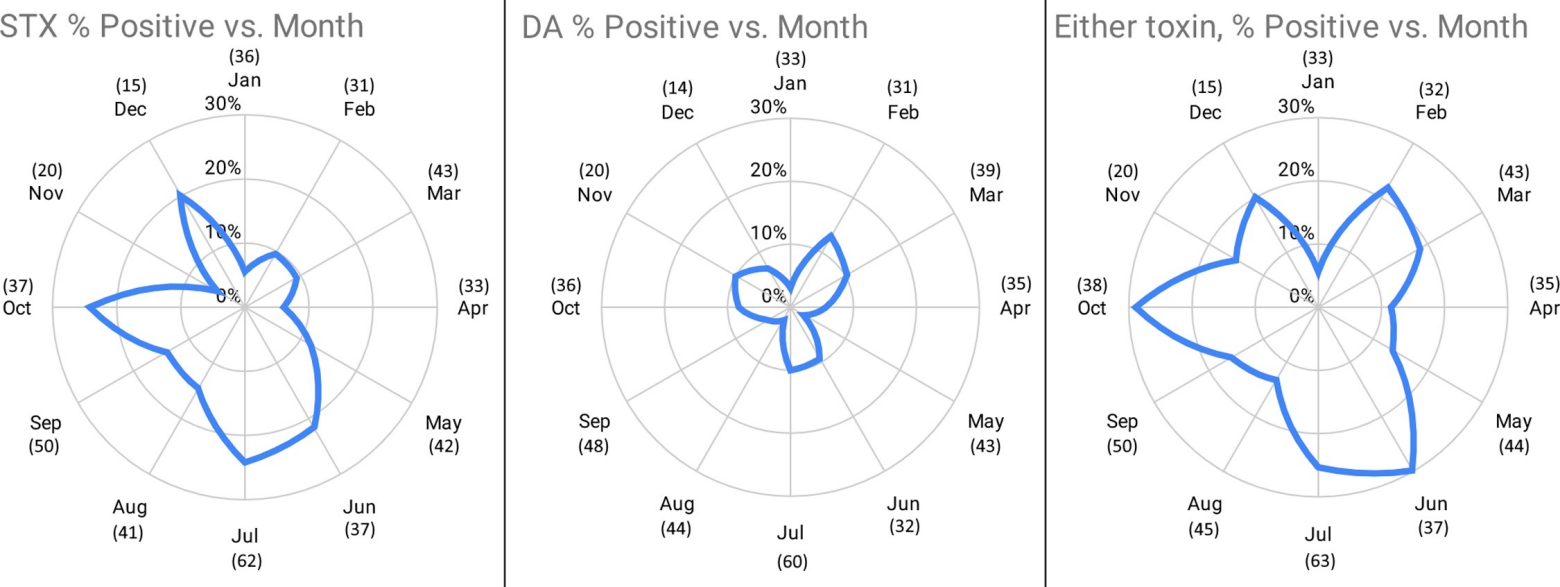
Frequency of animals testing positive for saxitoxin, domoic acid or either toxin, by month.

The percentage of animals testing positive for STX varied by month, and ranged from 5% to 24% (Fig 6). The proportion of STX-positive animals was lowest during January, and highest for the months of June, July and October, with between 22–24% animals stranding during those months testing positive for STX in one or more samples. The frequency of occurrence of STX-positive animals during January was significantly lower relative to July (p = 0.031) and October (p = 0.039), with January animals being 5.43 and 5.46 times less likely to test STX-positive, respectively. The percentage of animals testing positive for DA also varied by month of stranding, ranging from 2% to 13%. The highest proportion of DA-positive animals occurred during February (13%), followed by March (10%), July (10%) and November (10%). However, differences in frequencies of DA-positive animals by month were not statistically significant.

**Fig 7:**
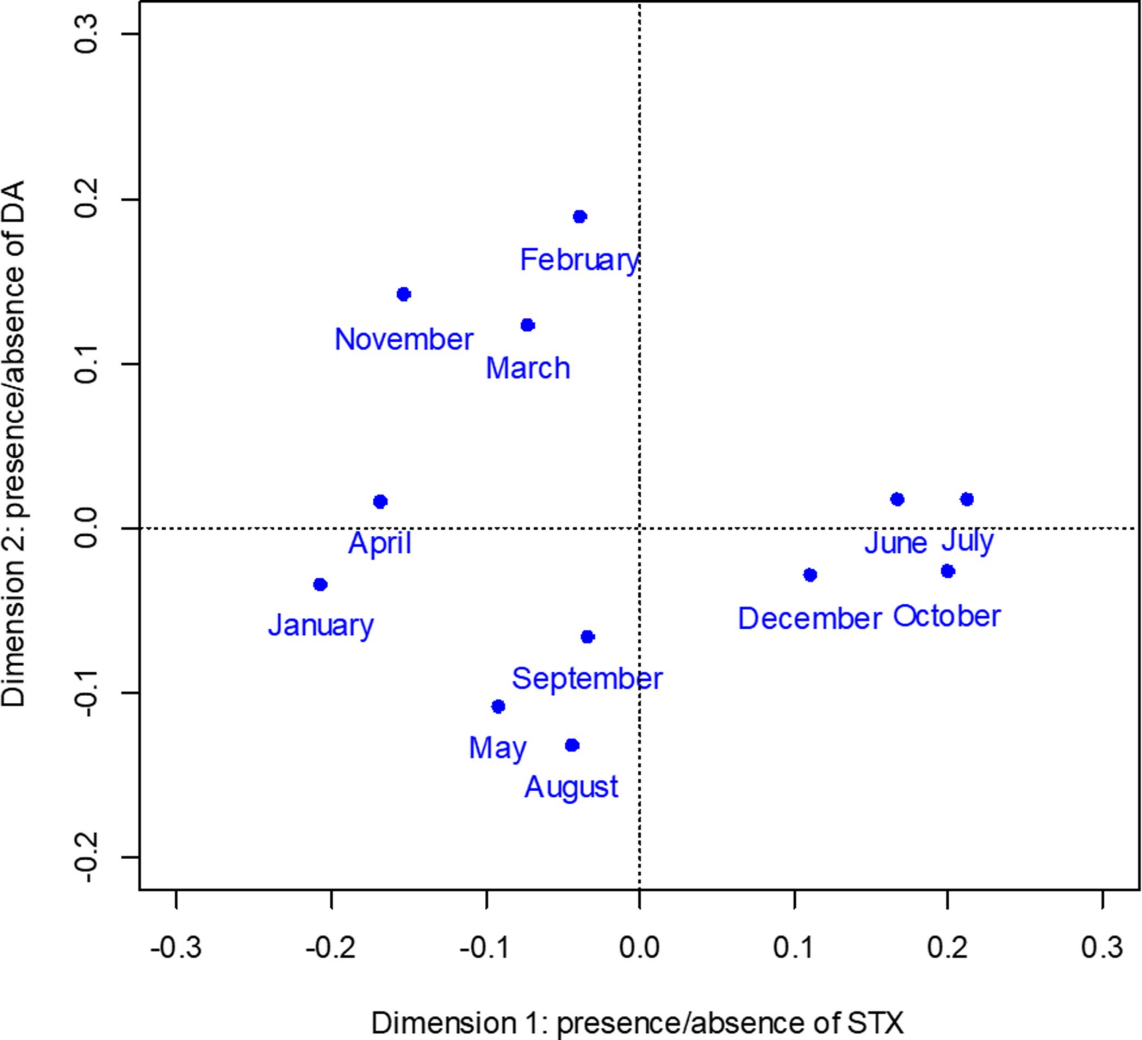
Correspondence analysis ordination for frequency comparisons by month of sampling. Positive values for dimension 1 correlate with larger proportion of positive saxitoxin samples and negative values correlate with larger proportion of negative saxitoxin samples. Positive values for dimension 2 correlated with larger proportion of positive domoic acid samples and negative values correlate with larger proportion of negative domoic acid samples.

CA dimension 1 explains 69.1% of the total inertia between toxins and month and correlates with STX positive/negative, 0.961 and 0.765 respectively. STX positive has the highest contribution, 795‰, to dimension 1. CA dimension 2 explains 28.3% of the total inertia between toxins and month and correlates with DA positive/negative 0.781 and 0.571 respectively. DA positive has the highest contribution, 786‰, to dimension 2. Table 9 summaries the correlations and contributions of each month. The CA ordination shows more separation among months in dimension 1 compared to dimension 2. The CA ordination also displays months with increasing instances of STX positive from left to right and increasing instances of DA positive from bottom to top (Fig 7).

**Table 9:** Month correlations and contributions to correspondence analysis dimensions 1 and 2.

	_Dimension 1		Dimension 2	
	Correlation	Contribution (‰)	Correlation	Contribution (‰)
January	0.927	0.165	0.025	0.011
February	0.040	0.005	0.934	0.297
March	0.249	0.024	0.718	0.169
April	0.952	0.107	0.009	0.002
May	0.419	0.040	0.578	0.134
June	0.918	0.107	0.011	0.003
July	0.990	0.304	0.007	0.005
August	0.095	0.009	0.855	0.201
September	0.197	0.006	0.757	0.058
October	0.980	0.161	0.017	0.007
November	0.533	0.052	0.459	0.109
December	0.919	0.019	0.059	0.003

**Fig 8:**
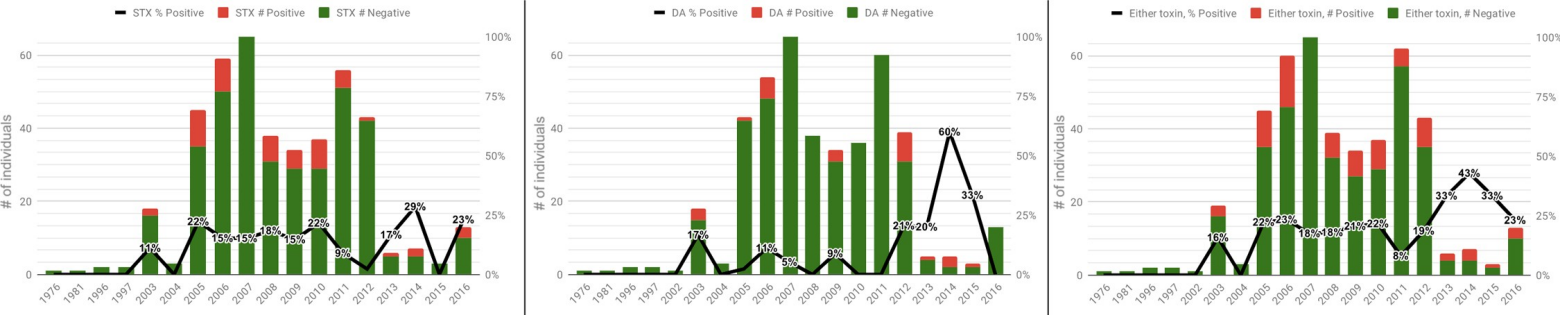
Animals testing positive for saxitoxin, domoic acid, or either toxin, by year.

Overall, the year in which animals were sampled was not a statistically significant predictor of the likelihood of testing positive for HAB toxins (Fig 8). For the entire dataset (all years included), there was no significant effect of year on frequencies of animals positive for DA (p = 0.201), STX (p = 0.874), or either toxin (p = 0.486). When excluding animals sampled prior to 2002 from the analysis to more accurately represent the bulk of the dataset, again no statistically significant effect was observed for STX (p = 0.363), DA (p = 0.277), or either toxin (p = 0.949).

For comparisons of toxin-positive animals between bloom seasons, 16.7% (36 of 215) of DBS individuals were STX-positive, compared to 12.5% (29 of 232) of NBS animals. However, no statistically significant difference in frequencies of STX-animals between DBS and NBS groups was found (p > 0.205). Although DA bloom season categories were not defined for this study, performing a similar analysis with the assumption that the DA bloom season is also from April-August also found no significant difference between DBS and NBS detection frequencies for DA (p > 0.507) or for either toxin (p > 0.731).

## Discussion

Overall, our data suggest that marine mammal populations in the coastal New England region of the United States experience frequent and possible repeated exposure to one or more HAB toxins, and this exposure varies significantly in time and space, and with taxonomic grouping. Here we address each aspect in turn and discuss the possible implications of our findings for each type of analysis. In total, approximately 1 out of every 5 animals in our study demonstrated evidence of exposure to one or more HAB toxins. STX and DA were present in 15% and 7% of animals tested, respectively, and in several instances (2%), both toxins were present in the same individual.

### Geographic trends

While HABs producing STX and DA are common throughout the New England coast, our findings suggest that STX exposure in marine mammals is concentrated in the northern portion of our study region. The majority of animals tested (93%) were from ME and MA, however ME animals were nearly twice as likely to test STX-positive relative to MA animals. This is consistent with results of long-term phytoplankton distribution surveys demonstrating that STX-producing *Alexandrium fundyense* blooms originate in the Maine/Bay of Fundy region to the north, and are transported alongshore to the southwest (NH, MA) via the Maine Coastal Current [56, 57]. It is also in agreement with data from shellfish toxicity monitoring efforts demonstrating that the corresponding impacts of STX in the food web in this region is likewise concentrated in the northern portion of the Gulf of Maine [53, 58]. The distribution of DA in marine mammals showed a somewhat reversed geographical trend. In decreasing order, OB animals had the highest proportion of DA-positive individuals (30%), followed by MA (8%) and ME (3%). Although long-term trends of DA-producing blooms in our study region are unclear, especially for offshore locations where there is limited monitoring, our findings are supported by previous work indicating widespread DA contamination of shellfish beds throughout the Georges Bank region during 2004–2007 [59]; NOAA, unpub.).

Since the physiological consequences of mammalian exposure to DA and STX are similar (neurological dysfunction and locomotor impairment), it is also worth considering the combined frequencies of toxin exposure in our sample set. In decreasing order, 35% of OB animals tested positive for either toxin, followed by ME (20%) and MA (16%). Although the proportion of toxin-positive animals was significantly greater for OB animals, it is not known whether this is a result of greater HAB activity in the offshore habitats where they were sampled, or a result of being caught alive, healthy and possibly actively feeding on toxin-contaminated prey until immediately prior to death. One possible explanation is that since STX and DA are both highly toxic, water soluble molecules that can completely depurate from mammalian systems within several hours [60], stranded animals may be exposed to lethal toxin levels that impair feeding and locomotion but are mostly eliminated via urine and feces during the intervening hours prior to death. In this scenario, decreased toxin concentrations in stranding animals would result in lower frequency of detection relative to bycaught animals. Regardless of the conditions surrounding premortem exposure, these findings suggest that HAB toxin exposure for New England marine mammals at least superficially follows the known geographic distribution of STX and DA (biological sources and/or food web sinks) in the same region. It should be noted however, that the distribution of animal sampling locations in this study is not necessarily an accurate reflection of the spatial use of these animals prior to death. Some of the species included are nearshore coastal residents of their respective ranges, but many others are pelagic animals with wide distributions, and may have had significant postmortem transport via coastal currents prior to stranding [61]. Additional follow-up investigation is necessary before translating these results to entire stocks for management purposes.

### Taxonomic and dietary trends

Our findings indicate no association between taxonomic group and probability of STX exposure, nor was there a significant relationship between dietary preference and STX exposure. This suggests that when STX is present in New England marine mammal habitats and/or feeding grounds, the toxin is equally accessible to mysticetes, odontocetes and pinnipeds, possibly due to widespread distribution throughout their respective food webs. However, on the species level, *D*. *delphis* was STX-positive 12 to 16 times more often relative to *H*. *grypus*, *B*. *acutorostrata*, *M*. *novaeangliae*, *P*. *groenlandicus*, *P*. *vitulina*, and *G*. *melas*. This indicates that, in spite of broad STX availability in the food web, some prey species may act as toxin vectors that disproportionately concentrate STX in their tissues relative to other prey items [2, 62]. For example, during several HAB-associated marine mammal MMEs in California and Florida, small, schooling, planktivorous fish such as anchovy (*Engraulis mordax*) or menhaden (*Brevoortia* sp.) were highly abundant in the stomach contents of the animals sampled, with greatly elevated concentrations of HAB toxins in their tissues [6, 18, 38]. In addition, recent investigations of long-term trends of HAB toxin exposure in southern California cetaceans also indicate a relationship between *D*. *delphis*, HAB toxin exposure, and a diet consisting primarily of planktivorous fish like *E*. *mordax* (Danil et al., in review). In terms of DA exposure, mysticetes tested positive more often than odontocetes, which in turn tested positive more often relative to phocids. By species, *M*. *novaeangliae* tested positive several times more often than *P*. *vitulina* and *P*. *groenlandicus*. These differences in DA exposure may be due to prey choice (feeding on lower-trophic level organisms), however this is not supported by our analyses of this dataset, which found no significant differences in DA-positive frequencies between prey groups. An alternative explanation for the taxonomic differences in STX or DA detection may be a result not of exposure, but of toxin residence time and toxicokinetics in the exposed organism. Even among experimentally-dosed mammalian models, STX and DA both exhibit high variability in the clearance time via urinary or fecal excretion routes [63, 64]. This in turn may be a result of differences in metabolic rates between taxonomic groups or species, with further study needed prior to making these conclusions.

### Temporal trends of HAB toxin exposure

As the presence of STX- or DA-producing blooms is often closely coupled to seasonal trends of daylight length and nutrient availability, we tested whether the corresponding toxin exposure in marine mammals reflected this seasonality. July and October were found to have significantly higher STX detection rates when excluding all other factors, and that the lowest frequency of toxin detection occurred during January. For July and January, this corresponds well to general phytoplankton bloom dynamics in the northern hemisphere, when summer months typically have greater primary productivity and bloom frequency and winter months are restricted by increased water column mixing and reduced insolation [65, 66]. It also corresponds to the observed bloom seasonality for *Alexandrium* sp. in the Gulf of Maine (May-August), however we found no significant differences in STX-detection frequencies between our DBS and NBS groups. This is likely due to the effect of HAB toxins necessarily having to pass through multiple organisms and trophic levels prior to marine mammal exposure, each with differential rates of accumulation, detoxification and depuration. As for DA exposure, neither month nor season appeared to have a relationship with likelihood of detection.

There is much emerging concern that HABs globally are increasing in frequency and severity, and as coastal oceans continue to warm due to climate change, the range of HAB species will expand and result in increased ecological impacts [67, 68]. This predicted increase is expected to in turn affect marine mammal food webs and frequency of detection of HAB toxins in marine mammal tissues [69]. However, when grouped by year, we found no overall association between time of sampling and probability of testing positive for either STX or DA. In addition, in spite of the severe Gulf of Maine red tide event and co-occurring marine mammal MMEs during 2005–2006, there was similarly no significant difference in STX detection frequency between these two years and the remaining years in our study period (p = 0.220). It should be noted, however, that this and the above temporal analyses of HAB toxin detections were based on a dataset where available samples were not equally represented over time. The bulk of samples were collected between 2005–2012, a time frame which is unsuitable for testing the corresponding long-term trends in oceanographic processes which typically are measured over decades [70].

### Distribution of toxin in body compartments

As a methodological practicality rather than part of an ecological investigation, we also compared the likelihood of various sample types testing positive for STX or DA. Since marine mammal stranding response is often a logistical and budgetary challenge, time and funds available for sample collection is often a limiting factor in determining the role of HAB toxins in marine mammal mortalities. In such cases, not all body compartments relevant for toxicological analysis can feasibly be sampled, and testing a full suite of samples from each individual can be costly and/or redundant when analyzing multiple animals from the same mortality event. Thus, it becomes necessary to prioritize which sample types will yield the best representation of toxin exposure, based on their likelihood of testing positive. Our findings indicate that for both STX and DA, feces and urine samples tested positive with greater frequency relative to other sample matrices, and should therefore be collected as top priority samples for future HAB-associated stranding response. It should be noted, however that this priority recommendation only holds true for STX and DA, since other HAB toxins exist (e.g. PbTx, DSP toxins) that are lipid-soluble and have a higher affinity and accumulation in the liver and fatty tissues [71, 72].

### Comparison of exposure relative to other regions

**Fig 9:**
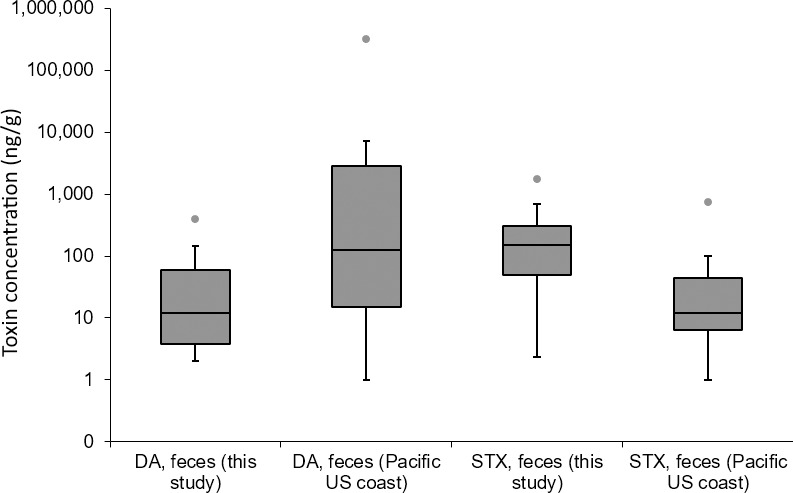
Domoic acid and saxitoxin fecal concentrations (ng/g) detected in this study vs. previously reported values in Pacific U.S. marine mammals. Box lines correspond to 25%, 50% and 75% quartiles, whisker lines correspond to 1.5 times the interquartile range, circle markers represent outliers beyond 1.5 times the interquartile range.

In terms of the degree of HAB toxin exposure for individual animals, relative comparisons can be made to other HAB-associated stranding events in the available literature, using STX or DA concentrations in the same sample matrices. Our finding of feces as the most useful indicator for DA and STX exposure in New England marine mammals is consistent with previous studies investigating impacts of HABs on marine mammals from other regions (Fig 9). Fecal DA concentrations detected in the present study (median 12, max. 394 ng/g) were approximately an order of magnitude less than those reported for various marine mammal species from the Pacific US coast (median 127, max. 324,000 ng/g) where HAB-related MMEs are frequent and severe [4, 17, 18, 38, 73–80]; S. Fire, unpub. data). This is possibly a result of more common upwelling events along the Pacific coast combined with the dominance of phytoplankton species (*Pseudo-nitzschia* spp.) that produce greater amounts of DA relative to their less-toxic counterparts along the US Atlantic coast [81, 82]. In contrast, fecal STX values in the present study (median 152, max. 1790 ng/g) were approximately an order of magnitude greater than those reported for various marine mammal species along the Pacific coast (median 12, max. 755 ng/g), where STX is routinely detected throughout the food web but has not been directly responsible for large MMEs [17]; S. Fire, unpub. data).

## Conclusions

Overall, our findings suggest that STX and DA are both commonly detected in New England marine mammals and are widespread across geographic locations, species and time scales. Since nearly all HAB toxin data for marine mammals is from dead-stranded animal response, it generally remains unknown what concentration thresholds of HAB toxins are required to induce morbidity and mortality. It is encouraging however, that compared to other regions of the coastal U.S. where HAB-associated marine mammal MMEs are frequent and severe, the New England region has had far fewer documented cases of similar events. Despite this, the health impacts on living marine mammals frequently exposed to one or more toxins is entirely unknown for this region, and warrants further investigation. The impacts of repeated, sublethal DA exposure in pinnipeds from the Pacific coast include reproductive failure and a chronic epileptic syndrome, but it is not known whether these impacts are also manifest in New England marine mammal species [73, 83]. In addition, *in vitro* studies using pinniped immune cells have demonstrated that exposures to low levels of STX and DA within the ranges reported here increase the likelihood of systemic viral infection or white blood cell proliferation [84, 85]. It is therefore incumbent on future related work to incorporate studies of longer-term health effects on living survivors of New England HABs. To that end, this dataset and the findings reported here establish reference values for future investigations of HAB-associated marine mammal mortalities, particularly on an individual-animal basis where determining cause of death is a priority. Having reference values for frequencies of toxin detection by sub-region or species aids resource managers in determining the involvement of HABs during future MMEs, should they occur in this area. Additionally, the Gulf of Maine is the fastest-warming water body on earth, with sea surface temperatures over the last decade increasing faster than 99% of the global ocean [86]. Increasing ocean temperatures in the North Atlantic have already been a major facilitating factor in the intensification of blooms of multiple HAB species in the region, including the STX producer *A*. *fundyense* [87]. Therefore, investigations continuing to assess ecosystem health by monitoring important sentinel species such as marine mammals benefit from these baseline reference points as a method of tracking the impacts of global climate change on critical habitats.

## Supporting information

S1 Table Data summary for animal collection data and toxin analytical results. (XLSX)
